# Development and Validation of Digital Decision Aids to Support Medication Adherence in Older Adults: A Scoping Review

**DOI:** 10.3390/pharmacy14060129

**Published:** 2026-09-02

**Authors:** Ghada Elba, Shaheena Fakir, Rishabh Sharma, Halak Patel, Caitlin Carter, Tejal Patel

**Affiliations:** 1 School of Pharmacy, University of Waterloo, Waterloo, ON N2G 1C5, Canada; gelba@uwaterloo.ca (G.E.); shaheena.fakir@uwaterloo.ca (S.F.); r367sharma@uwaterloo.ca (R.S.); h398pate@uwaterloo.ca (H.P.); c8carter@uwaterloo.ca (C.C.); 2 Schlegel-UW Research Institute for Aging, Waterloo, ON N2J 0E2, Canada

**Keywords:** decision aid, older adults, digital, medication adherence, validation

## Abstract

Introduction: The development of technology to support medication management and adherence among older adults is rising. Many of these technologies offer diverse features, which add complexity when making a choice. Evidence-based decision aids can inform decision-making in such situations. To inform the development of such a decision aid, it is necessary to review the literature on the development and validation of decision aids that impact medication adherence. Therefore, this study aims to review the available literature on digital decision aids that examine the impact on medication adherence and investigate their features, development, and validation processes. Methods: Adopting the Arksey and O’Malley scoping review framework, a comprehensive search was conducted using PubMed, Embase, CINAHL, IPA and Scopus, using four key concepts: medication adherence, decision aids/tools, older adults, and software/technology. Eligibility criteria included studies that enrolled older adults with a mean/median age of 65 years, published protocols for studies that supported decision-making, published in the English language, and examined and reported the development and/or validation and/or evaluation of digital decision aids. Results: Our search identified 3526 records. After removing 947 duplicates, 2579 records underwent title and abstract screening, of which 726 proceeded to full-text screening. Following full-text screening, one research protocol met all eligibility criteria and was included in the review. An additional 16 systematic reviews were examined to identify potentially relevant studies from their reference lists; however, none of the studies identified through this supplementary screening met the eligibility criteria. Conclusion: The limited number of eligible studies shows a substantial gap in the literature on the development and validation of digital decision aids that support medication adherence among older adults. Further research in this field is needed to develop and validate evidence-based and user-centered digital decision aids for this population.

## 1. Introduction

The population of adults aged 65 years and older has been increasing in Canada [1]. According to Statistics Canada, adults aged 65 years and older numbered approximately 7.6 million in 2023, accounting for 18.9% of the Canadian population, compared to 2013, when older adults accounted for 15.3% of the population [1]. This proportion of the population is expected to grow more in the coming decade, reaching 10.4 million by 2037 [2]. While the growth of the population of older adults is the result of advanced technology [3], improvement of healthcare [4], and social determinants of health [5], aging is frequently accompanied by an increase in challenges [6]. One example of such a challenge is the increased risk of comorbidity, i.e., having multiple chronic illnesses at the same time [7,8,9].

Chronic diseases require long-term treatment with multiple medications [10]. Polypharmacy, defined as taking five or more medications at the same time [7,8], is prevalent among older adults [8,11]. Although management of chronic diseases with the appropriate medication is important, polypharmacy can lead to medication regimen complexity, medication errors and medication non-adherence [10].

Medication adherence is defined as ”the extent to which a person’s behavior—taking medication, following a diet, and/or executing lifestyle changes—corresponds with agreed recommendations from a health care provider” [12]. Medication non-adherence can lead to suboptimal disease management, hospitalization, financial burden, and even death [13,14,15]. One of the interventions for addressing medication non-adherence is the use of medication adherence technologies. Such technologies support medication management at home by offering different features, like medication organization, portability, automated reminders, real-time monitoring of medication intake, locking features, and automatic dispensing, among others [16,17,18,19]. Examples of such technologies include automated medication dispensers that dispense medication at the time of the dose, electronic pill boxes with sensors that record the time of opening to facilitate real-time monitoring of medication dispensation, smart blister packaging that can connect to a cloud for recording and storage of medication dispensation data, and ingestible sensors that can record accurate ingestion of the drug [18].

Although the use of medication adherence technologies has demonstrated a promising impact on improving medication adherence [16,20], the variety of features they offer may make it challenging to choose the right product. Information on the usability of the different features of the medication adherence technologies is necessary to support older adults making informed decisions when choosing a product to support their medication-taking behavior [19,21,22]. Furthermore, the usability of medication adherence technologies will differ among individuals who have different physical, sensory, and cognitive abilities. For example, audible alarms may be ineffective in individuals with loss of hearing. For such individuals, visual and sensory alarms may be more appropriate [23,24]. The usability of technologies is a key factor for their effectiveness at achieving their purpose, ensuring user satisfaction and continued use [25,26]. Therefore, it is important to provide such information to support decision-making related to the choice of medication adherence technology.

Given the myriad features available with medication adherence technologies [27], a decision aid can guide the selection of the most appropriate medication adherence technology. Decision aids streamline decision-making based on the users’ specific characteristics, preferences and needs by providing evidence-based information about the available options, associated benefits, harm, probabilities, and scientific uncertainties [28]. According to a systematic review, studies have shown that decision aids improve alignment between an informed patient’s preferences and the chosen option [29]. Decision aids enhance the involvement of patients in decision-making and increase comfort with their choices by improving their knowledge of options and their perceptions of outcome probabilities [29]. This is of significant importance because an increase in knowledge and appreciation for risk often motivates people to change their initial decisions [29].

While numerous decision aids exist for the management of medical conditions, surgical options, and choice of nursing homes, among others, [30], there are no decision aids that guide in the choice of the most appropriate medication adherence technology. We aim to develop such a decision aid. To guide the development of this decision aid, we conducted a scoping review to examine the literature that details the processes used by other researchers to develop and/or validate digital decision aids aimed at supporting medication adherence in older adults. In addition, we aimed to examine the features of such decision aids to guide the design of our decision aid for medication adherence technologies.

## 2. Materials and Methods

The scoping review was conducted based on the Arksey and O’Malley scoping review framework [31], which consists of 5 stages:

Stage 1: Identifying the research question.

Stage 2: Identifying relevant studies. 

Stage 3: Selecting the studies.

Stage 4: Charting the data. 

Stage 5: Collating, summarizing, and reporting the results. 

The details of this scoping review are being reported according to the PRISMA extension for scoping reviews (PRISMA-ScR) [32].

### 2.1. Stage 1: Identify the Research Question+

The two research questions being addressed in this scoping review are:

1. “What are the processes (theoretical frameworks, development methodologies, step-by-step processes) used to develop and/or validate digital decision aids that support medication adherence in older adults?” We aimed to identify the steps used in creating, developing, and validating decision aids to guide the development of our own decision aid.
2. What are the features of such decision aids? We aimed to abstract data on design features (for example, color, contrast, format of presentation of data), the presentation of options, and the presentation of evidence for comparison.

We defined “digital” as an interactive tool characterized by electronic and/or computerized technology that utilizes user inputs to generate personalized solutions [33]. We defined a decision aid based on the Ottawa Decision Support Framework (ODSF). According to the ODSF, a decision aid is a tool that guides users in making decisions. Decision aids streamline decision-making based on the users’ specific characteristics, preferences, values and needs by providing evidence-based information about the available options, associated benefits, harms, probabilities, and scientific uncertainties. Decision aids promote a comparison of pros and cons of behavior changes. This process is often referred to as decision balance. To support decision-making, users are often provided with decisional balance worksheets to aid in the comparison of costs, challenges, or negative outcomes to benefits, rewards or positive outcomes of different options [34].

We defined “processes” as methodologies/procedures using a series of steps to achieve a certain goal or outcome, including information needed, actions taken, and output [35]. Similarly, we defined “development” as the process required to create or produce, especially by deliberate effort over time, i.e., by starting from nothing or just a simple tool to a more advanced and refined one [36]. And finally, “validation” was defined as demonstrating or supporting the truth or value of something [37].

### 2.2. Stage 2: Identifying Relevant Studies

Five databases were searched including: PubMed (Medline); Ovid Embase; Cumulative Index to Nursing and Allied Health Literature (CINAHL) via EBSCOhost; Ovid International Pharmaceutical Abstracts (IPA); and Scopus. The search strategy concepts included medication adherence, decision aids/tools, older adults, and software/technology. For each concept, a set of keywords and controlled vocabulary were determined and incorporated into the search strategy using Boolean operators (AND/OR), truncation, and adjacency/proximity operators, where appropriate. All database search strategies were limited to the English language only. The relevant studies were then exported into Covidence^®^ (Veritas Health Innovation, Melbourne, Australia) for screening. The identification of the search strategy keywords and controlled vocabulary was conducted with the help of a research librarian (C.C.) and is detailed in Supplementary Material S1.

### 2.3. Stage 3: Study Selection

After the database search results were exported into Covidence^®^, duplicates were removed. Following that, all remaining records were screened by pairs of reviewers (G.E., S.F., R.S., and T.P.) such that each title and abstract and each full-text article was reviewed by two reviewers independently. The first round of screening was limited to titles and abstracts. Those publications that met eligibility criteria (Table 1) or ones where eligibility was not possible to determine from abstract review were included in the second round of screening. Conflicts were resolved through a discussion between reviewers on a weekly basis. Screening and full-text review was conducted systematically by first determining if the tool was a decision aid, followed by determining the mean/median age of the included sample, then by type of population of interest (older adults or caregivers), then if the tool was digital, and finally by whether the study measured medication adherence as an outcome. Any phase of medication adherence was considered eligible, including initiation of new drugs, persistence with ongoing treatment and discontinuation of instructions provided. Any method of measurement of medication adherence was included.

To ensure we identified all the relevant literature, we reviewed systematic reviews identified in our literature review for the purpose of reviewing the included studies to determine eligibility for our scoping review. The reviewers screened the articles included in each systematic review by applying the same eligibility criteria.

### 2.4. Stage 4: Charting the Data and Stage 5: Collating, Summarizing and Reporting the Results

Data charting and extraction were completed by two reviewers (G.E. and H.P.) independently. Extracted variables included: title, authors, study location, year of publication, aim, study design, sample size, age, gender, sex, race, income, education, place of living, social status, decision aid tool components, frameworks used, steps used in designing and/or developing decision aids, validation and/or evaluation methods, tools and methods of measurement of adherence and other outcomes. Data extraction was completed using Microsoft^®^ Excel^®^ (Office 365 Version 2406). The reviewers discussed the abstracted data for any conflicts, which were resolved upon discussion by reaching an agreement.

## 3. Results

### 3.1. Article Selection

**Figure 1:**
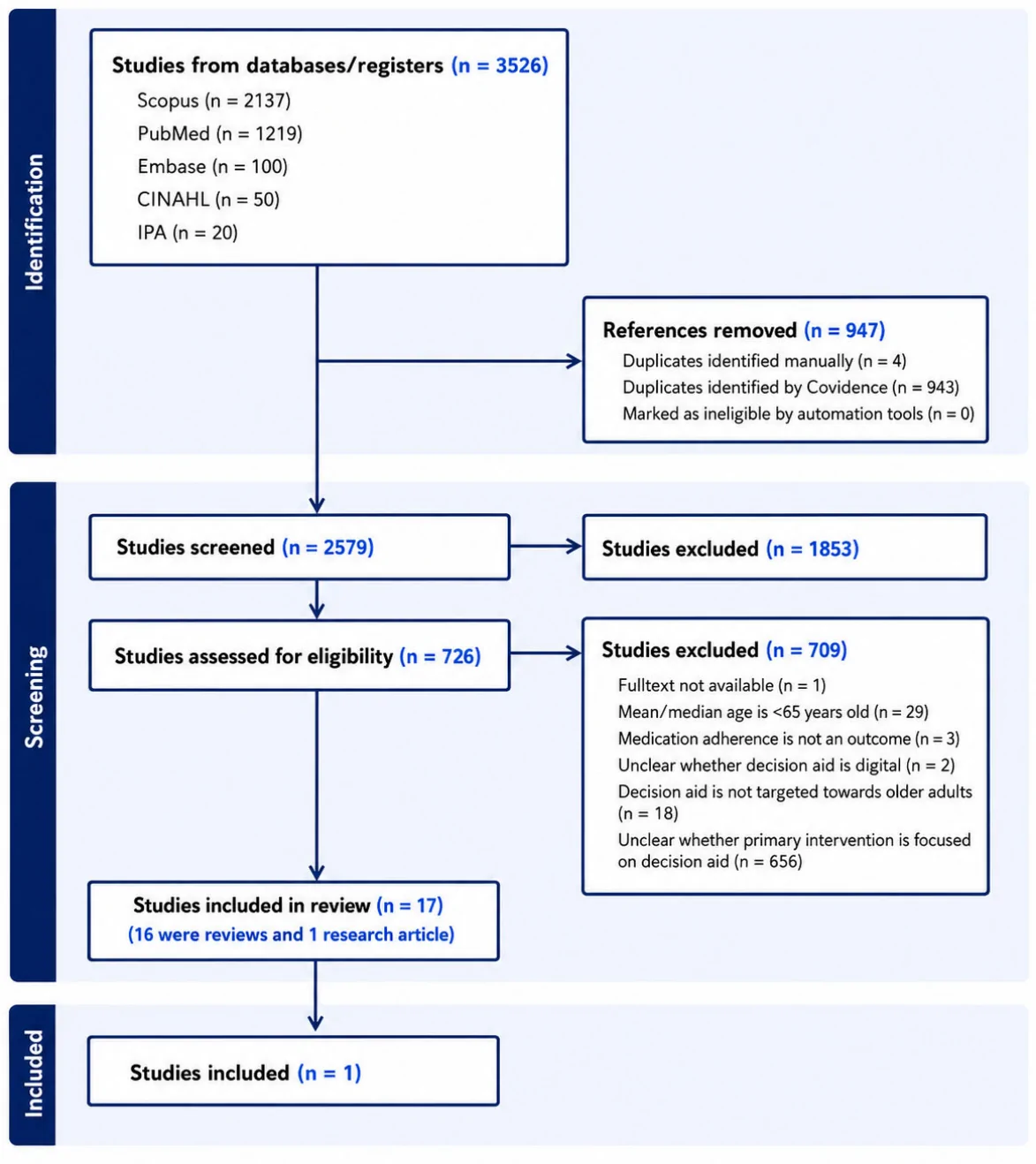
PRISMA diagram for retrieved and extracted articles.

Our database search yielded 3526 articles. After 947 duplicates were removed, 2579 articles were included for title and abstract screening (see Figure 1). Of these articles, 726 articles were included for full-text screening and review. Following full-text screening, 17 publications were retained for further assessment: one research protocol that met our eligibility criteria and 16 systematic reviews that were subjected to an additional level of review. We further reviewed and screened the studies included in these 16 systematic reviews to identify studies that met our eligibility criteria. However, none of the articles identified from the systematic reviews met the eligibility criteria.

### 3.2. Included Article

The only included publication that met our eligibility criteria was a research protocol published by Langdon et al. [38]. Table 2 shows the characteristics and planned development and evaluation processes of this study.

The main objective of this protocol was to develop, refine, and evaluate the acceptability and feasibility of an interactive computer and text message-based behavioral therapy intervention to support buprenorphine induction and stabilization, which was called iCOPE. iCOPE incorporates decisional balance personalized feedback to support distress tolerance training skills among individuals who use buprenorphine for addiction treatment. The decisional balance was expected to optimize motivation for making a behavioral change by displaying the advantages and disadvantages of abstinence from opioids and using buprenorphine, as well as providing strategies to manage emotional and physical discomfort associated with this behavior change.

This protocol outlines the use of the NIH Stage Model [39] to guide the development of the intervention and the decisional balance tool for the use of buprenorphine. This protocol describes Stage 1 of the six-stage model, which the authors further divided into Stage 1A and 1B. In Stage 1A, the steps the researchers describe to develop and refine their intervention include conducting qualitative interviews to investigate the proposed content of iCOPE, technology use, and its facilitators and barriers. Moreover, investigators will refine iCOPE through open pilot testing, in which they will evaluate the comprehensibility and appropriateness of the intervention, along with prior experience with similar interventions, perceived usefulness, their preferences, the likelihood of continued use, approval of the format and content, and any suggestions for improving the tool. Finally, feasibility will be assessed by recruitment rates, the proportion completing the intervention, study attrition and follow-up rates, while usability and acceptability will both be assessed using the System Usability Scale (SUS) and the Relative Subjective Count (RSC). RSC is the participant’s estimate of the number of times the system delivers a text, divided by the number of texts delivered.

In stage 1B, authors will pilot test the effectiveness of the intervention through a randomized controlled trial where the outcome of interest is medication adherence. Medication adherence will be measured through self-reported daily use of opioids, illicit substances and alcohol, as well as through urine analysis.

## 4. Discussion

The goal of this scoping review was to identify detailed processes used in the development and validation of digital decision aids, and the features of such decision aids that could be used by older adults and impact medication adherence. Despite an extensive `search, only one publication met our eligibility criteria. This finding indicates that there is a paucity of literature that outlines the development and/or validation of digital aids that can support older adults in making decisions that impact medication adherence.

To better understand this limited evidence base, we examined the principal reasons for exclusion and representative examples. Of the 726 publications assessed through full-text screening, 709 were excluded. The majority of these publications (n = 656; 92.5% of all full-text exclusions) were excluded because it was unclear whether the primary intervention focused on a decision aid or a decision-making process. Several publications used terms such as “interactive”, “person-centered”, “self-management”, “shared decision-making”, or “decision support”; however, the interventions did not clearly identify a specific decision or provide a structured process through which users could compare available options according to their values and preferences.

For example, Andersson et al. evaluated an interactive, web-based hypertension self-management system that collected blood pressure, medication intake, symptoms, and lifestyle information and provided reminders, motivational messages, and graphical feedback [40]. Although shared decision-making was discussed, the digital system did not explicitly present or compare treatment options or elicit users’ values and preferences. Similarly, two publications by Anglada-Martínez et al. described the development and evaluation of Medplan, a web- and smartphone-based medication self-management platform [41,42]. Medplan provided medication reminders, medication information, adherence monitoring, motivational messages, and patient–professional communication. However, it did not clearly identify a preference-sensitive decision or provide a structured comparison of alternative options.

Twenty-nine publications were excluded because the mean or median age of the study population was below 65 years. For example, Adarkwah et al. evaluated the computerized Arriba cardiovascular decision aid in the Optimizing Risk Communication trial [43]. Although eligible participants were aged 30–80 years, the mean age was 58.24 years in the emoticon-based risk presentation group and 58.20 years in the time-to-event group. Therefore, the study did not meet the prespecified mean or median age threshold of 65 years or older.

Eighteen publications were excluded because the target group for the decision support intervention was healthcare clinicians. For example, Accorsi et al. evaluated an International Classification of Diseases code-triggered prescription decision support system used by physicians during urgent telemedicine consultations [44]. The study included patients aged 16 years or older and was not designed specifically for older adults. Furthermore, the system supported clinicians by automatically populating guideline-based prescriptions rather than supporting older patients in making medication-related decisions.

Three publications were excluded because medication adherence was not measured as an outcome. For example, Rahn et al. described POWER@MS2, an interactive, web-based decision aid supporting relapse management decisions among people with multiple sclerosis [45]. Its primary outcome was the proportion of relapses that were untreated or treated with oral corticosteroids. References to adherence to the intervention concerned participants’ engagement with the online program rather than their adherence to prescribed medications.

Two publications were excluded because it was unclear whether the evaluated decision aid met our operational definition of a digital intervention. For example, Bortolussi-Courval et al. described MedSafer, an electronic system that generated individualized deprescribing recommendations from patient medication and health information entered into a web-based portal [46]. However, the reports were generated in advance, printed, placed in a study binder, and delivered to the clinical team during medication reconciliation. Patients received printed deprescribing brochures. Thus, although the recommendations were generated electronically, clinicians and patients did not interact directly with the digital system during the decision-making process. Finally, the full text of one publication could not be obtained.

Collectively, these exclusions demonstrate methodological and terminological inconsistencies in the literature. Interactivity, self-management support, or the use of electronic technology does not necessarily make an intervention a digital decision aid. Future studies should clearly identify the intended users, the decision being supported, the available options, the values clarification process, the method of personalization, the digital delivery format, and the medication adherence outcome being measured. Clear reporting of these elements would facilitate the identification and comparison of digital decision aids in future reviews.

Within this limited evidence base, the protocol by Langdon et al. [38] provides an example of a staged approach to developing and validating a digital intervention incorporating a decisional balance component. In this protocol [38], the authors used the NIH Stage Model [39] as their guiding framework for developing their decision-making tool. This framework is a staged iterative model, which is commonly used in developing interventions focused on behavioral change. It consists of six stages: investigating basic sciences (informing stage), intervention generation and refinement, testing efficacy in research settings, testing efficacy in community settings, testing effectiveness (external validation), and implementation and dissemination.

In this protocol, although the decision to use buprenorphine, likely a key component of iCOPE intervention, was guided by decisional balance in the intervention, it is unclear whether the NIH Stage Model is appropriate for the development of a decision aid, as the model was developed to guide the development of a behavioral intervention. In contrast, a well-known and referenced decision aid development, evaluation and implementation framework is the International Patient Decision Aid Standards (IPDAS) [47]. IPDAS provides best practices for developing effective and high-quality decision aids. The stepwise process recommended by IPDAS includes (1) identification of the scope of the decision aid; (2) establishing a steering committee to guide the development; (3a) assessing patient and clinician decision needs; (3b) determining the format and distribution plan; (3c) reviewing and synthesizing evidence; (4) drafting a prototype; (5) conducting an alpha test with end users for comprehensibility, acceptability and feasibility; (6) iteration based on alpha testing results; (7) and finally, beta testing for usability before final implementation.

To display the decision-making information about the available choices in the iCOPE tool, the authors will employ the decisional balance approach, in which they will display the advantages and disadvantages of all options. This aligns with one of the IPDAS domains, “providing balanced information” [48]. Balanced information about the options is essential to educate the users and guide their informed decisions [47,48]. The decisional balance approach was also used by Hollen et al. [49] in their decision aid tool “Decision-KEYS for Balancing Choices: Cancer Care”, which was targeted towards patients with advanced cancer who have challenges making high-quality and informed decisions about their treatment options. In this study, the participants found the tool both acceptable and feasible. Moreover, another study conducted by Geller et al. [50] tested the implementation of a decisional balance sheet to promote behavior changes in diet and physical activity among older adults. In this study, the decisional balance program was both effective and efficient in changing behaviors in this population.

In stage 1A of the iCOPE development, the authors will utilize inductive and deductive approaches to capture users insights and needs during qualitative interviews; this approach will allow comprehensive and in-depth analysis of their ideas and preferences by integrating both new data-driven insights and previous knowledge to inform the development of a user-centered tool [51]. This process is very similar to steps 3 and 4 of the IPDAS framework. For the development of our digital decision aid, using both data analysis approaches will help us comprehensively capture the needs of older adults in a decision aid that offers the advantages and disadvantages of different medication adherence technologies.

Stage 1B, as outlined by Langdon et al., is closely aligned with steps 5 and 6 of the IPDAS framework. While Langdon et al. are planning on conducting a randomized controlled pilot testing of their iCOPE intervention, we intend to examine the acceptability, appropriateness and usability in iterative alpha and beta testing stages with older adults, care partners and clinicians. While this study aims to validate their decision aid with a randomized controlled trial where the outcome is medication adherence, other researchers have utilized different validation methodologies. For example, Hild et al. [52] used the Delphi consensus method to validate the content of their decision aids. Experts rated the content and design, site navigation and readability of the decision aid, and evaluated them on a Likert scale. The tool was then refined based on these findings and insights. These examples highlight that our decision aid validation could be accomplished using multiple methodologies, including self-reported questionnaires, expert consensus methods and end-user evaluation. Weighing the pros and cons of each method along with their feasibility in terms of time, cost and practicality would guide our choice.

A key finding was the lack of studies that can guide the development of digital aids for older adults. Designing a digital tool for older adults has to consider the following factors, as outlined by Jeff Johnson [53]: motor and vision challenges, hearing and speech needs, cognitive requirements, knowledge presentation and their attitude towards using the tool. Some design guidelines to accommodate those factors may include adopting a familiar and simple design, including graphical language whenever possible, positioning icons in accessible locations on the screen, maximizing the legibility of the essential information, ensuring obvious feedback when clicking an object, allowing speech input and output, adding an accessibility mode, consistency in design and terms used, using user-friendly language, and finally, ensuring an appealing design and flexible navigation. To ensure the digital format of our decision aid is usable by older adults, we will conduct mixed-methods studies in steps 3 to 6 of the IPDAS framework.

**Figure 2:**
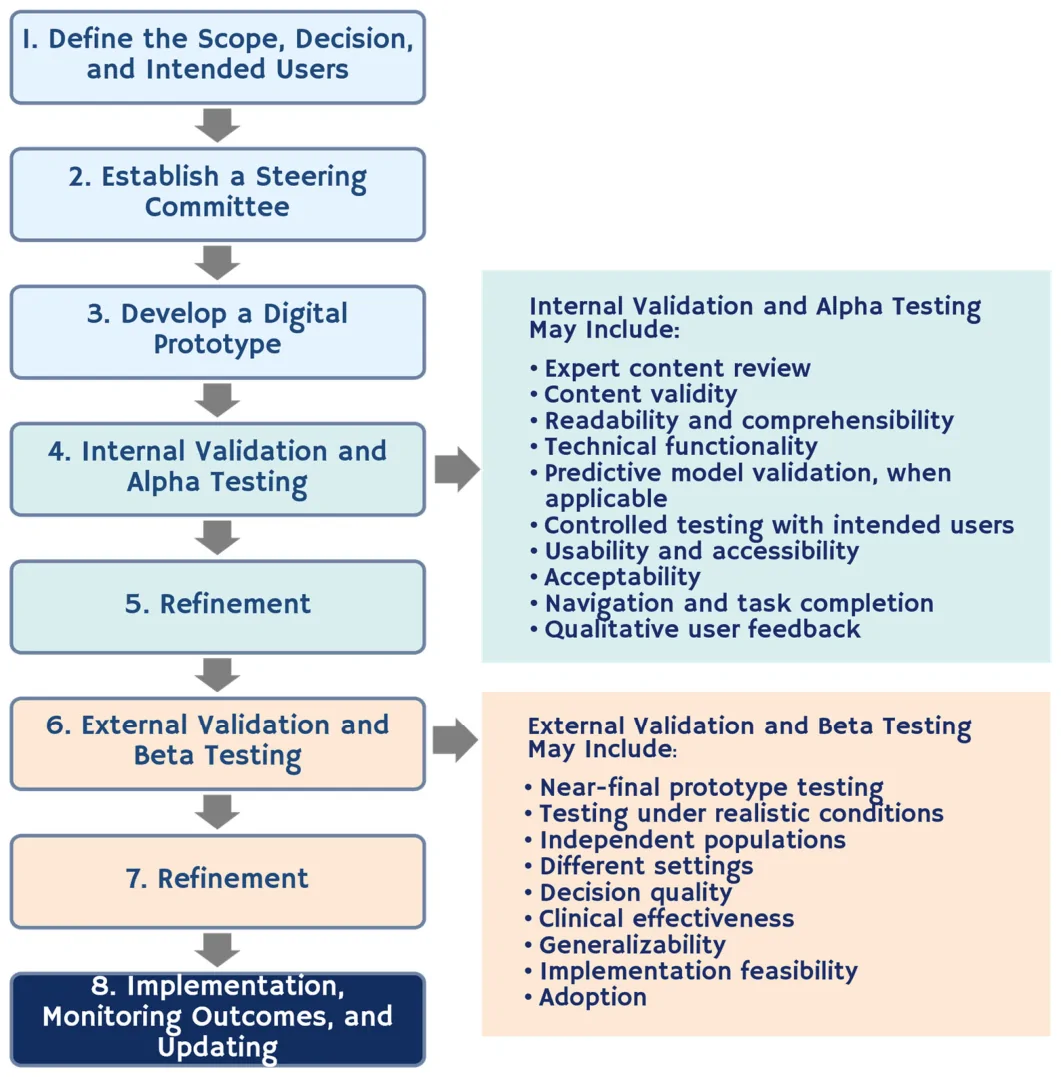
Proposed eight-stage pathway for developing, validating, implementing, and updating digital decision aids for medication adherence among older adults, informed by the NIH Stage Model, IPDAS, and design recommendations for older adults [39,47].

Based on the NIH Stage Model, IPDAS, and design recommendations for older adults, we propose an eight-stage pathway for developing and validating digital decision aids that support medication adherence among older adults (Figure 2) [39,47]. The pathway covers defining the decision and intended users, stakeholder co-design, prototype development, internal validation and alpha testing, refinement and beta testing, external validation, and implementation.

### 4.1. Study Limitations

Some of the limitations we had in our scoping review include restricting our search to the English language, which could have led to the exclusion of relevant articles in other languages. Additionally, due to feasibility and time constraints, we did not conduct our search in the grey literature, which may have limited the identification of some eligible decision aids and reports on their development and validation. Our strict eligibility criteria may also have excluded publications containing transferable evidence on co-design, usability, decision quality, and validation, including non-digital or clinician-facing decision aids and studies involving younger populations. Although these exclusions may have narrowed the range of development and validation approaches identified, they were necessary to maintain the focus of the review on digital decision aids supporting medication adherence among older adults. Finally, our results are limited to February 2025, so later studies may not be included in our review.

### 4.2. Future Directions

The one eligible publication identified in this scoping review highlights the importance of directing future research towards developing more digital decision-making guides targeted toward older adults to support medication adherence. The findings from this scoping review, along with using the IPDAS framework, will guide our development process of user-friendly digital decision aids that support older adult decision-making on medication adherence technologies.

## 5. Conclusions

In conclusion, this scoping review establishes a gap in digital decision aids that were designed for older adults to measure the impact on medication usage. By directing future research on developing user-centered, accessible and evidence-based decision aids, healthcare systems will be better equipped to support older adults in making health-related decisions and, therefore, achieving better healthcare outcomes and improved quality of life.

## Figures and Tables

**Table 1:** Eligibility criteria.

Inclusion Criteria	Exclusion Criteria
Age mean/median of 65 years or older Target users of decision aids are older adults Published in the English language Examined and reported the development and/or validation and/or evaluation of decision aids Reported on the development of decision aids in digital format	Studies in which the decision aid is not targeted towards older adults Paper-based decision aids Theses, book chapters, letters, commentaries, or conference abstracts Unclear whether primary intervention is focused on decision aid Medication adherence not measured as an outcome

**Table 2:** Characteristics and planned development and evaluation processes of the included study.

Characteristic	Description
Study	Langdon et al. (2020) [38]
Title	Development of an Integrated Digital Health Intervention to Promote Engagement in and Adherence to Medication for Opioid Use Disorder
Country and setting	United States; Lifespan Recovery Center, an outpatient addiction treatment center at Rhode Island Hospital
Publication type	Study protocol
Study purpose	To develop and refine iCOPE and examine its feasibility, acceptability, and preliminary efficacy in improving abstinence, adherence to buprenorphine, and treatment retention
Target population	Adults initiating outpatient buprenorphine treatment for opioid use disorder
Participant age	18–75 years in the planned randomized controlled trial (mean age not available, as this is a protocol)
Planned sample	Stage 1A: 24 qualitative interview participants and 16 additional open-pilot participants; Stage 1B: 80 participants randomized to iCOPE or treatment as usual at a 3:1 ratio
Intervention	iCOPE, an interactive personalized feedback intervention combining motivational enhancement with distress-tolerance skills training
Digital delivery format	One brief computer-delivered session followed by eight weeks of personalized and automated text messages
Decision support component	Personalized decisional balance exercise presenting the advantages and disadvantages of abstaining from opioids and adhering to buprenorphine treatment; the resulting information is used to provide personalized motivational feedback
Development framework	Stage I of the NIH Stage Model/NIDA Behavioral Therapies Development Program
Stage 1A: development and refinement	Qualitative interviews examining intervention content, technology use, treatment barriers and facilitators, perceived usefulness, and preferences; inductive and deductive analysis; iterative intervention refinement; and open pilot testing
Stage 1B: preliminary evaluation	Pilot randomized controlled trial comparing iCOPE with treatment as usual; assessments at baseline and 1, 4, 8, and 12 weeks after treatment initiation
Feasibility assessment	Recruitment and refusal rates, intervention completion, follow-up rates, and study attrition
Usability and acceptability assessment	System Usability Scale, Relative Subjective Count, participant satisfaction, perceived usefulness, comprehensibility, appropriateness, likelihood of continued use, and qualitative feedback
Primary outcome	Percentage of days positive for opioid, other illicit substance, or alcohol use, measured using self-report and urine toxicology
Medication adherence assessment	Percentage of days of buprenorphine administration, assessed through self-report and urine toxicology
Other outcomes	Treatment retention, motivation, distress tolerance, emotional and behavioral self-regulation, and negative affect
Study status and limitation	Protocol publication; development, feasibility, acceptability, usability, and effectiveness results were not yet reported

Abbreviations: iCOPE, integrated computer- and text message-delivered personalized feedback intervention; NIDA, National Institute on Drug Abuse; NIH, National Institutes of Health.

## Data Availability

No new data were created or analyzed in this study. Data sharing is not applicable to this article.

## Supplementary Materials

The following supporting information can be downloaded at: https://www.mdpi.com/article/10.3390/pharmacy14060129/s1, Supplementary Material S1: Search strategies.
