# Supplementary material for: Development and Validation of Digital Decision Aids to Support Medication Adherence in Older Adults: A Scoping Review
Source: Pharmacy (Basel). 2026 Sep 2;14(6):129. doi: 10.3390/pharmacy14060129 (PMC13610242; doi:10.3390/pharmacy14060129)
Supplement: Supplementary file 1 [file pharmacy-14-00129-s001.zip › pharmacy-4437474-supplementary.pdf]

**Identifying digital decision aids used by older adults for medication adherence:
A scoping review.**

Supplementary Material S1: Search strategies

PubMed(MEDLINE) search strategy:

(Decision Support Techniques[mesh] OR Surveys and questionnaires[mesh] OR Psychometrics[mesh] OR Decision Making[mesh] OR Decision Making, Computer-Assisted[mesh] OR “Decision aid*” OR “Decision guide*” OR Tool* OR Instrument* OR Framework* OR Assess* OR Survey* OR Questionnaire* OR Guide* OR checklist* OR IPDAS OR Evaluat* OR Scale* OR Screen* OR Measur* OR Psychometric* OR impact* OR support* OR “Decision making”[tiab:~2] OR “Decisions making”[tiab:~2] OR “Make decisions”[tiab:~2] OR “Decision support technic*” OR “Decision support model*” OR “Decision modeling” OR “Decision analys*” OR “Decision process”[tiab:~4] OR “Decision processes”[tiab:~4] OR “Decisions process”[tiab:~4] OR “Decisions processes”[tiab:~4]) AND (Medication Adherence[mesh] OR “Medication adherence”[tiab:~4] OR “Medications adherence”[tiab:~4] OR “Medication adhering”[tiab:~4] OR “Medications adhering”[tiab:~4] OR “Drug adherence”[tiab:~4] OR “Drugs adherence”[tiab:~4] OR “Drug adhering”[tiab:~4] OR “Drugs adhering”[tiab:~4] OR “Medication non-adherence”[tiab:~4] OR “Medications non-adherence"[tiab:~4] OR “Medication non-adhering”[tiab:~4] OR “Medications non-adhering"[tiab:~4] OR “Medication nonadherence”[tiab:~4] OR “Medications nonadherence”[tiab:~4] OR “Medication nonadhering”[tiab:~4] OR “Medications nonadhering”[tiab:~4] OR “Drug non-adherence”[tiab:~4] OR “Drugs non-adherence"[tiab:~4] OR “Drug non-adhering”[tiab:~4] OR “Drugs non-adhering"[tiab:~4] OR “Drug nonadherence”[tiab:~4] OR “Drugs nonadherence”[tiab:~4] OR “Drug nonadhering”[tiab:~4] OR “Drugs nonadhering”[tiab:~4] OR “Medication compliance”[tiab:~4] OR “Medications compliance”[tiab:~4] OR “Drug compliance”[tiab:~4] OR “Drugs compliance”[tiab:~4] OR “Medication non-compliance”[tiab:~4] OR “Medications non-compliance"[tiab:~4] OR “Medication noncompliance”[tiab:~4] OR “Medications noncompliance”[tiab:~4] OR “Drug non-compliance”[tiab:~4] OR “Drugs non-compliance"[tiab:~4] OR “Drug noncompliance”[tiab:~4] OR “Drugs noncompliance”[tiab:~4] OR “Medication persistence”[tiab:~4] OR “Medications persistence”[tiab:~4]) AND (Software[mesh] OR Mobile Applications[mesh] OR telemedicine[Mesh] OR Software* OR “Computer program*” OR “Computer application*” OR “Mobile application*” OR “mobile health application*” OR “Mobile app” OR “Mobile apps” OR Smartphone* OR “Smart phone*” OR “Assistive technolog*” OR “Assistive device*” OR “Assistive tool*” OR ehealth* OR telemedicine OR telehealth* OR “Computer assisted” OR digital*) AND (Aged[mesh] OR Aged OR Elder* OR “Older people*” OR “Older person*” OR “Older male*” OR “Older female*” OR “Older man” OR “Older woman*” OR “Older women” OR “Older men” OR “Senior citizen*” OR Geriatric* OR “Older population*” OR “Older patient*” OR Septuagenarian* OR Octogenarian* OR Octagenarian* OR Nonagenarian* OR Centenarian*) AND English[lang]

Ovid Embase search strategy

.	exp decision support system/
2.	exp health care survey/
3.	exp health survey/
4.	exp questionnaire/
5.	exp psychometry/

6.	exp decision making/
7.	exp checklist/
8.	("Decision aid*" or "Decision guide*" or Tool* or Instrument* or Framework* or Assess* or Survey* or Questionnaire* or Guide* or checklist* or IPDAS or Evaluat* or Scale* or Screen* or Measur* or Impact* or Support* or Psychometric*).mp. [mp=title, abstract, heading word, drug trade name, original title, device manufacturer, drug manufacturer, device trade name, keyword heading word, floating subheading word, candidate term word]
9.	(decision* adj2 making).mp. [mp=title, abstract, heading word, drug trade name, original title, device manufacturer, drug manufacturer, device trade name, keyword heading word, floating subheading word, candidate term word]
10.	(make adj2 decision*).mp. [mp=title, abstract, heading word, drug trade name, original title, device manufacturer, drug manufacturer, device trade name, keyword heading word, floating subheading word, candidate term word]
11.	("Decision support technic*" or "Decision support model*" or "Decision modeling" or "Decision analys*").mp. [mp=title, abstract, heading word, drug trade name, original title, device manufacturer, drug manufacturer, device trade name, keyword heading word, floating subheading word, candidate term word]
12.	(decision* adj4 process*).mp. [mp=title, abstract, heading word, drug trade name, original title, device manufacturer, drug manufacturer, device trade name, keyword heading word, floating subheading word, candidate term word]
13.	1 or 2 or 3 or 4 or 5 or 6 or 7 or 8 or 9 or 10 or 11 or 12
14.	exp medication compliance/
15.	((drug* or medication*) adj4 (adher* or nonadher* or "non-adher*" or compliance or noncompliance or "non-compliance")).mp. [mp=title, abstract, heading word, drug trade name, original title, device manufacturer, drug manufacturer, device trade

	name, keyword heading word, floating subheading word, candidate term word]
16.	(medication* adj4 persistence).mp. [mp=title, abstract, heading word, drug trade name, original title, device manufacturer, drug manufacturer, device trade name, keyword heading word, floating subheading word, candidate term word]
17.	14 or 15 or 16
18.	exp software/
19.	exp mobile application/
20.	exp telehealth/
21.	(Software* or "Computer program*" or "Computer application*" or "Mobile application*" or "mobile health application*" or "Mobile app*" or "Smartphone*" or "Smart phone*" or "Assistive technolog*" or digital or "Assistive device*" or "Assistive tool*" or "Computer assisted" or "ehealth*" or telemedicine or telehealth*).mp. [mp=title, abstract, heading word, drug trade name, original title, device manufacturer, drug manufacturer, device trade name, keyword heading word, floating subheading word, candidate term word]
22.	exp aged/
23.	(Aged* or Elder* or "Older people*" or "Older person*" or "Older male*" or "Older female*" or "Older man" or "older men" or "Older woman*" or "Older women" or "Senior citizen*" or Geriatric* or "Older population*" or "Older patient*" or Septuagenarian* or Octogenarian* or Octagenarian* or Nonagenarian* or Centenarian*).mp. [mp=title, abstract, heading word, drug trade name, original title, device manufacturer, drug manufacturer, device trade name, keyword heading word, floating subheading word, candidate term word]
24.	22 or 23
25.	13 and 17 and (Software* or "Computer program*" or "Computer application* or Mobile application* or mobile health application* or Mobile app* or

	Smartphone* or Smart phone* or Assistive technolog* or digital or Assistive device* or Assistive tool* or Computer assisted or ehealth*" or telemedicine or telehealth*).mp. and 24 [mp=title, abstract, heading word, drug trade name, original title, device manufacturer, drug manufacturer, device trade name, keyword heading word, floating subheading word, candidate term word]

EBSCOhost CINAHL search strategy

#	Query	Limiters/Expanders	Last Run Via	Results
S24	S11 AND S15 AND S19 AND S22	Expanders - Apply equivalent subjects<br>Search modes - Proximity	Interface - EBSCOhost Research Databases<br>Search Screen - Advanced Search<br>Database - CINAHL	3,861
S23	S11 AND S15 AND S19 AND S22	Expanders - Apply equivalent subjects<br>Search modes - Proximity	Interface - EBSCOhost Research Databases<br>Search Screen - Advanced Search<br>Database - CINAHL	3,861
S22	S20 OR S21	Expanders - Apply equivalent subjects<br>Search modes - Proximity	Interface - EBSCOhost Research Databases<br>Search Screen - Advanced Search<br>Database - CINAHL	Display
S21	(Aged OR Elder* OR “Older people*” OR “Older person*” OR “Older male*” OR “Older female*” OR “Older man” OR “older men” OR “Older woman” OR “Older women” OR “Senior citizen*” OR Geriatric* OR “Older population*” OR “Older patient*” OR Septuagenarian* OR Octogenarian* OR Octagenarian* OR	Expanders - Apply equivalent subjects<br>Search modes - Proximity	Interface - EBSCOhost Research Databases<br>Search Screen - Advanced Search<br>Database - CINAHL	Display

	Nonagenarian* OR Centenarian*)			
S20	(MH "Aged+")	Expanders - Apply equivalent subjects<br>Search modes - Proximity	Interface - EBSCOhost Research Databases<br>Search Screen - Advanced Search<br>Database - CINAHL	Display
S19	S16 OR S18	Expanders - Apply equivalent subjects<br>Search modes - Proximity	Interface - EBSCOhost Research Databases<br>Search Screen - Advanced Search<br>Database - CINAHL	647,496
S18	(Software* OR “Computer program*” OR “Computer application*” OR “Mobile application*” OR “mobile health application*” OR “Mobile app*” OR Smartphone* OR “digital” OR “Smart phone*” OR “Assistive technolog*” OR “Assistive device*” OR “Assistive tool*” OR “Computer assisted”)	Expanders - Apply equivalent subjects<br>Search modes - Proximity	Interface - EBSCOhost Research Databases<br>Search Screen - Advanced Search<br>Database - CINAHL	628,285
S17		Expanders - Apply equivalent subjects<br>Search modes - Proximity	Interface - EBSCOhost Research Databases<br>Search Screen - Advanced Search<br>Database - CINAHL	0
S16	(MH "Software+") OR (MH "Mobile Applications") OR (MH "telehealth")	Expanders - Apply equivalent subjects<br>Search modes - Proximity	Interface - EBSCOhost Research Databases<br>Search Screen - Advanced Search<br>Database - CINAHL	Display
S15	(S12 OR S13 OR S14)	Expanders - Apply equivalent subjects<br>Search modes - Proximity	Interface - EBSCOhost Research Databases<br>Search Screen - Advanced Search<br>Database - CINAHL	62,509
S14	medication* N4 persistence	Expanders - Apply equivalent subjects<br>Search modes - Proximity	Interface - EBSCOhost Research Databases<br>Search Screen - Advanced	418

			Search<br>Database - CINAHL	
S13	(drug* OR medication*) N4 (adher* OR nonadher* OR “non-adher*” OR compliance OR non-compliance OR “noncompliance”)	Expanders - Apply equivalent subjects<br>Search modes - Proximity	Interface - EBSCOhost Research Databases<br>Search Screen - Advanced Search<br>Database - CINAHL	30,766
S12	(MH "Medication Compliance") OR (MH "Compliance with Medication Regimen (Saba CCC)") OR (MH "Compliance with Medical Regimen (Saba CCC)") OR (MH "Patient Compliance+")	Expanders - Apply equivalent subjects<br>Search modes - Proximity	Interface - EBSCOhost Research Databases<br>Search Screen - Advanced Search<br>Database - CINAHL	Display
S11	(S1 OR S2 OR S3 OR S4 OR S5 OR S6 OR S7 OR S8 OR S9 OR S10)	Expanders - Apply equivalent subjects<br>Search modes - Proximity	Interface - EBSCOhost Research Databases<br>Search Screen - Advanced Search<br>Database - CINAHL	4,194,479
S10	(MH "Research Instruments+")	Expanders - Apply equivalent subjects<br>Search modes - Proximity	Interface - EBSCOhost Research Databases<br>Search Screen - Advanced Search<br>Database - CINAHL	Display
S9	(“Decision support technic*” OR “Decision support model*” OR “Decision modeling” OR “Decision analys*”)	Expanders - Apply equivalent subjects<br>Search modes - Proximity	Interface - EBSCOhost Research Databases<br>Search Screen - Advanced Search<br>Database - CINAHL	Display
S8	decision* N4 process*	Expanders - Apply equivalent subjects<br>Search modes - Proximity	Interface - EBSCOhost Research Databases<br>Search Screen - Advanced Search<br>Database - CINAHL	14,987
S7	make N2 decision*	Expanders - Apply equivalent subjects<br>Search modes - Proximity	Interface - EBSCOhost Research Databases<br>Search Screen - Advanced Search<br>Database - CINAHL	Display
S6	decision* N2 making	Expanders - Apply equivalent subjects<br>Search modes - Proximity	Interface - EBSCOhost Research Databases<br>Search Screen - Advanced	Display

			Search<br>Database - CINAHL	
S5	“Decision aid*” OR “Decision guide*” OR Tool* OR Instrument* OR Framework* OR Assess* OR Survey* OR Questionnaire* OR Guide* OR checklist* OR IPDAS OR Evaluat* OR Scale* OR Screen* OR Measur* OR Psychometric* OR support* OR impact*)	Expanders - Apply equivalent subjects<br>Search modes - Proximity	Interface - EBSCOhost Research Databases<br>Search Screen - Advanced Search<br>Database - CINAHL	4,070,200
S4	(MH "Decision Making, Patient+") OR (MH "Decision Making, Computer Assisted+") OR (MH "Decision Making+")	Expanders - Apply equivalent subjects<br>Search modes - Proximity	Interface - EBSCOhost Research Databases<br>Search Screen - Advanced Search<br>Database - CINAHL	Display
S3	(MH "Surveys+") OR (MH "Structured Questionnaires") OR (MH "Open-Ended Questionnaires")	Expanders - Apply equivalent subjects<br>Search modes - Proximity	Interface - EBSCOhost Research Databases<br>Search Screen - Advanced Search<br>Database - CINAHL	Display
S2	(MH "Decision Support Techniques+") OR (MH "Decision Support Systems, Management")	Expanders - Apply equivalent subjects<br>Search modes - Proximity	Interface - EBSCOhost Research Databases<br>Search Screen - Advanced Search<br>Database - CINAHL	Display
S1	(MH "Psychometrics")	Expanders - Apply equivalent subjects<br>Search modes - Proximity	Interface - EBSCOhost Research Databases<br>Search Screen - Basic Search<br>Database - CINAHL	Display

SCOPUS search strategy

(TITLE-ABS-KEY(("Decision aid*" OR "Decision guide*" OR tool* OR instrument* OR framework* OR assess* OR survey* OR questionnaire* OR guide* OR checklist* OR ipdas OR evaluat* OR scale* OR screen* OR measur* OR support* OR impact* OR psychometric*)) OR TITLE-ABS-KEY((decision* W/2 making)) OR TITLE-ABS-KEY((make W/2 decision*)) OR TITLE-ABS-KEY(("Decision support technic*" OR "Decision support model*" OR "Decision modeling" OR "Decision analys*")) OR TITLE-ABS-KEY((decision* W/4 process*)) AND TITLE-ABS-KEY((drug* OR medication* W/4 adher* OR nonadher* OR "non-adher*" OR compliance OR noncompliance OR "non-compliance")) OR TITLE-ABS-KEY((medication* W/4 persistence))

AND TITLE-ABS-KEY((software* OR "Computer program*" OR "Computer application*" OR "Mobile application*" OR "mobile health application*" OR digital OR "Mobile app*" OR smartphone* OR "Smart phone*" OR "Assistive technolog*" OR "Assistive device*" OR "Assistive tool*" OR "Computer assisted" OR ehealth* OR telemedicine OR telehealth*)) AND TITLE-ABS-KEY((aged* OR elder* OR "Older people*" OR "Older person*" OR "Older male*" OR "Older female*" OR "Older man" OR "Older woman*" OR "Older women" OR "Older men" OR "Senior citizen*" OR geriatric* OR "Older population*" OR "Older patient*" OR septuagenarian* OR octogenarian* OR octagenarian* OR nonagenarian* OR centenarian*))) AND (LIMIT-TO (LANGUAGE,"English"))

Ovid International Pharmaceutical Abstracts search strategy

Set	Search Statement	Annotations	Insert	Edit
1.	("Decision aid*" or "Decision guide*" or Tool* or Instrument* or Framework* or Assess* or Survey* or Questionnaire* or Guide* or checklist* or IPDAS or Evaluat* or Scale* or Screen* or Support* or Impact* or Measur* or Psychometric*).mp. [mp=title, subject heading word, registry word, abstract, trade name/generic name]			
2.	(decision* adj2 making).mp. [mp=title, subject heading word, registry word, abstract, trade name/generic name]			
3.	(make adj2 decision*).mp. [mp=title, subject heading word, registry word, abstract, trade name/generic name]			
4.	("Decision support technic*" or "Decision support model*" or "Decision modeling" or "Decision analys*").mp. [mp=title, subject heading word, registry word, abstract, trade name/generic name]			
5.	(decision* adj4 process*).mp. [mp=title, subject heading word, registry word, abstract, trade name/generic name]			

6.	or/1-5
7.	((drug* or medication*) adj4 (adher* or nonadher* or "non-adher*" or compliance or noncompliance or "non-compliance")).mp. [mp=title, subject heading word, registry word, abstract, trade name/generic name]
8.	(medication* adj4 persistence).mp. [mp=title, subject heading word, registry word, abstract, trade name/generic name]
9.	or/7-8
10.	(Software* or "Computer program*" or "Computer application*” or “Mobile application*” or “mobile health application*” or “Mobile app*” or “Smartphone*” or “Smart phone*” or “Assistive technolog*” or “Assistive device*” or “Assistive tool*” or “Computer assisted” or digital" or ehealth* or telemedicine or telehealth*).mp. [mp=title, subject heading word, registry word, abstract, trade name/generic name]
11.	(Aged or Elder* or "older men" or "Older people*" or "Older person*" or "Older male*" or "Older female*" or "Older man" or "Older woman" or "Older women" or "Senior citizen*" or Geriatric* or "Older population*" or "Older patient*" or Septuagenarian* or Octogenarian* or Octagenarian* or Nonagenarian* or Centenarian*).mp. [mp=title, subject heading word,

	registry word, abstract, trade name/generic name]
12.	6 and 9 and 10 and 11
13.	limit 12 to english language
